# Validation of the P1vital® Faces Set for Use as Stimuli in Tests of Facial Emotion Recognition

**DOI:** 10.3389/fpsyt.2022.663763

**Published:** 2022-02-11

**Authors:** Julia A. Romano, Laura Vosper, Jonathan A. Kingslake, Colin T. Dourish, Suzanne Higgs, Jason M. Thomas, Andreea Raslescu, Gerard R. Dawson

**Affiliations:** ^1^P1vital Ltd., Wallingford, United Kingdom; ^2^P1vital Products Limited, Oxfordshire, United Kingdom; ^3^School of Psychology, University of Birmingham, Birmingham, United Kingdom; ^4^Department of Psychology, Aston University, Birmingham, United Kingdom

**Keywords:** psychiatric disease, depression, facial emotion recognition, P1vital® Affective Faces set, Pictures of Facial Affect

## Abstract

**Background:**

Negative bias in facial emotion recognition is a well-established concept in mental disorders such as depression. However, existing face sets of emotion recognition tests may be of limited use in international research, which could benefit from more contemporary and diverse alternatives. Here, we developed and provide initial validation for the P1vital® Affective Faces set (PAFs) as a contemporary alternative to the widely-used Pictures of Facial Affect (PoFA).

**Methods:**

The PAFs was constructed of 133 color photographs of facial expressions of ethnically-diverse trained actors and compared with the PoFA, comprised of 110 black and white photographs of facial expressions of generally Caucasian actors. Sixty-one recruits were asked to classify faces from both sets over six emotions (happy, sad, fear, anger, disgust, surprise) varying in intensity in 10% increments from 0 to 100%.

**Results:**

Participants were significantly more accurate in identifying correct emotions viewing faces from the PAFs. In both sets, participants identified happy faces more accurately than fearful faces, were least likely to misclassify facial expressions as happy and most likely to misclassify all emotions at low intensity as neutral. Accuracy in identifying facial expressions improved with increasing emotion intensity for both sets, reaching peaks at 60 and 80% intensity for the PAFs and PoFA, respectively. The study was limited by small sizes and age-range of participants and ethnic diversity of actors.

**Conclusions:**

The PAFs successfully depicted a range of emotional expressions with improved performance over the PoFA and may be used as a contemporary set in facial expression recognition tests.

## Introduction

The neural and psychological mechanisms underlying recognition of emotional expressions has been a subject of investigation for more than 100 years (1, 2). The literature on human social and emotional behavior is rich in studies of face processing and depends on availability of valid and reliable face stimuli balanced for factors such as gender and ethnicity (3). We describe a new set of facial expression stimuli developed for use in the P1vital® Facial Expression Recognition Task (FERT). This task displays faces that participants must categorize into one of six emotional categories based on their expression: happy, sad, fear, anger, disgust, surprise and neutral. The FERT has been the subject of considerable recent research interest since performance on the task has been shown to be influenced by subject moods (4). Low mood may lead a subject to interpret faces with ambiguous or neutral expressions as displaying a “negative” emotion, such as sadness, anger or fear.

Misattributions of emotion are thought to arise from a negative interpretative bias, central to depressive symptomatology. This view was first proposed by Beck et al. (5), who argued that a “cognitive triad” of negative views of the self, world and future can cause a negative distortion of environmental feedback in depressed individuals (6). According to this model, negative biases can automatically “hijack” cognition, creating a circle of negative thinking. As these biases become consistent, they may shift “default automatic processing” of affective information to create a stable, dysfunctional and self-reinforcing negative schemata (7). The main implication of the cognitive neuropsychological model is that pharmacological interventions do not affect mood directly. Instead, antidepressant drugs have been proposed to alter the brain's processing of affective stimuli early in the treatment process (8–10), allowing depressive symptoms to remit gradually as the contributing “bottom-up” biases are attenuated or abolished due to altered regulation of monoamine transmission. The possibility of assessing this response behaviorally significantly decreases research costs and allows implementation of large, randomized controlled clinical trials which can provide more conclusive evidence that early effects of antidepressant drugs can be used to predict response at the individual patient level, using a priori defined behavioral criteria (11).

However, such studies require well-validated, ecologically valid instruments for measuring changes in affective processing in large populations of depressed patients and non-depressed controls. For an instrument like the FERT, it is also important to have diverse, contemporary and novel sets of faces to facilitate international research studies. While the Pictures of Facial Affect (PoFA) produced in 1976 has been extensively validated for such research, it comprises black and white photos of generally Caucasian actors (12). For this reason, we developed the P1vital® Affective Faces set (PAFs) as a contemporary alternative, featuring 133 individual ethnically-diverse color photos of trained actors.

The current study aimed to identify equivalence between the PAFs and the PoFA in the FERT task as facial expression stimuli. For the purposes of this study, the tests were carried out using individuals with no history of mental illness. It was anticipated that this would help to minimize effects of negative emotional biases that might be observed using a depressed patient group. Thus, our main focus was to perform these tests in non-depressed controls to establish a baseline. The premise of the study is similar to that of an investigation by Mazurski and Bond (13), which compared participants' classifications of a set of Australian slides displaying facial expressions with those of the PoFA. Specifically, we investigated whether the participants' ability to recognize facial expressions varied between the PAFs and PoFA face sets, with the expectation that accuracy rates that are equivalent between the two face sets or favor the PAFs would provide initial empirical support for the validity of our novel face set. Furthermore, we hypothesized that the PAFs and PoFA would elicit similar response patterns from study participants in terms of accuracy to specific emotions, effects of emotion intensity and emotion misclassifications. This was based on previous studies suggesting a certain universality to the basic emotions of anger, disgust, fear, happiness, sadness, and surprise (14), and to the ease or difficulty with which they are recognized (15, 16).

As a secondary aim, we conducted exploratory analyses of interactions between participant and actor demographics, regarding gender and ethnicity. Although the study was not designed to specifically address these questions, the preliminary results could guide further validation studies of the PAFs in an increasingly international research space. There has now been extensive research on the effects of participant and actor race (17, 18) and gender (19, 20) on emotion recognition. No specific predictions were made with regards to data in this study, but modulation of recognition accuracy by participant and/or actor gender was expected.

Lastly, we investigated the potential relationship between participant Beck Depression Inventory (BDI) scores and recognition of positively and negatively-valenced facial stimuli in both image sets. Based on the results of the above studies, we predicted that higher BDI scores would be associated with reduced accuracy in identifying positive emotions, and an increased accuracy in identifying negative emotions in facial stimuli. Ultimately, our goal was to introduce a contemporary face set that can be used in studies of emotional processing, suitable for implementing in research with participants of varied age, gender or ethnic background.

## Methods

### Participants

Participants were recruited from Brunel University London and the University of Birmingham, and all received £10 reimbursement for their time. Data were collected from 61 participants between the ages of 18 and 45 years (Table 1). Approximately half of the participants were male and the majority were of White ethnicity (66%).

**Table 1:** Age, sex, ethnicity and years spent in formal education of study participants (*N* = 61), and breakdown of Facial Emotion Recognition Task (FERT) trials employing the P1vital® Affective Faces set (PAFs) according to actor sex and ethnicity.

		***N***	**%**
**Demographics**			
Age category	18–29 years	56	92
	30–39 years	3	5
	40–49 years	2	3
Sex	Male	31	51
	Female	30	49
Ethnicity	White	40	66
	Mixed	2	3
	Asian (non-Chinese)	5	8
	Black	9	15
	Chinese	3	5
	Other	2	3
Education	<11 years	0	0
	11 years	1	2
	13 years	15	24
	16 years	45	74
Total		61	100
**Emotion trials**			
Actor no.	Gender	Race	No. of trials shown (6 emotions x 10 intensities + 1 neutral)
Actor 1	Male	White	61
Actor 2	Female	Black British	61
Actor 3	Female	Asian (non-Chinese)	61
Actor 4	Female	Other	61
**Neutral trials**			
Actor no.	Gender	Race	No. of trials shown (1 neutral)
Actor 5	Male	White	1
Actor 6	Male	White	1
Actor 7	Female	White	1
Actor 8	Female	White	1
Actor 9	Female	White	1
Actor 10	Female	Black British	1

### Materials

Participants completed a questionnaire to collect basic demographic data, followed by the BDI questionnaire and two FERT tasks, employing either the PAFs or the PoFA. The FERT tasks were completed using a standard computer screen setup, with the faces presented in the middle of a 21.5 inch 1080p monitor on black background. Participants used specific response buttons to classify faces for each of the six possible emotions (happy, sad, fear, anger, disgust, surprise), and neutral.

### Beck Depression Inventory

Each participant completed the BDI (21), consisting of 21 questions generating a score between 0 and 63. This inventory has been used in over 500 published studies (17) and found to be correlated with clinician ratings of depression (22).

### Development of the PAFs

The development of the PAFs followed the methodology described by Tottenham et al. (3) for the NimStim Set of Affective Facial Expressions. In brief, 49 professional actors (12 male, 37 female) were trained to express anger, disgust, fear, happy, sad, surprise and neutral facial expressions, resulting in an initial set of 343 faces. Training for both datasets was conducted by specialists from the Paul Ekman Group using the Facial Action Coding System. Following training and accreditation that the actors in both datasets expressed emotions to 100% intensity, we systematically tested the faces for recognition accuracy using 190 online raters (male and female) and adjusted the morphed data set so that the accuracy resulted in similar scores by these participants. For each actor, 50% intensity was accurately identified by 50% of participants, 60% intensity was accurately identified by 60% of participants, etc. Thus, the sets were equivalent in terms of the how well each emotion for each actor was recognized at each intensity and morphed to be so. The ability of each actor to accurately express an emotion was assessed using the same 190 raters as above. To reduce test duration, images were divided into two equal datasets, A and B, matched by age, gender and ethnicity of the actors. The online raters were randomly allocated to one of the two groups and asked to identify the emotions expressed in images belonging to the corresponding dataset. Their choices were limited to one of the six emotions mentioned above, or neutral. Analysis of rating accuracy was conducted with SPSS (version 20.0). Mean accuracies for all emotions were above 70%, ranging from 75 to 99%, exceeding a previously set threshold of 60%. Following this analysis, the 19 actors whose emotional expressions were most accurately and readily identified were selected. Their images were morphed to create different levels of intensity for each emotion (10–100%, increasing in 10% increments), using specialized software (AbrosoftFantaMorph 5, 2012). This resulted in a final dataset of 1,159 images [(19 actors x 6 emotions x 10 intensities) + 19 neutral faces]. For this dataset four actors were selected from 19 actors to provide gender and ethnicity diversity (see Table 1). It consisted of 240 facial expressions each representing one of the six emotions at a particular intensity. Each emotion was included four times per intensity, as expressed by four different actors. An additional 10 neutral facial expressions (belonging to the same four actors mentioned above plus an additional six actors) were included, resulting in a face-set of 250 images (Table 1). The PoFA data set was identical to that extensively used by Harmer et al. (9). As with the PAF, four actors were selected from the Ekman and Friesen Pictures of Affect Series for gender and ethnicity diversity. This gave 240 facial expressions representing one of the six emotions at a particular intensity, with 10 additional neutral facial expressions as above to give a face-set of 250 total images.

### FERT Tasks

We employed a within-subjects design, with the order of the two FERT tasks counter-balanced. Each participant completed two FERT tasks, one employing the 250 facial expressions selected from the PAFs and an identical FERT using 250 facial expressions from the PoFA. Half of the participants completed the FERT employing PAF faces first, followed by the FERT employing PoFA faces. The other half completed the FERT tasks in the opposite order. During each FERT task, face stimuli were randomly presented on the screen for 500 ms, followed by a blank screen. The participant selected the emotion they believed was shown by pressing the corresponding button on a response box. Each FERT task took ~20 min to complete.

### Procedure

The study was approved by the ethical committees of Brunel University and the University of Birmingham. Study visits took place at both university campuses and lasted for ~60 min. Participants were given an information sheet and informed consent form to complete before taking part in the study. They were informed they could withdraw from the study at any point without penalty. Participants completed a short demographic questionnaire, the BDI and the two FERT tasks. As each facial stimulus appeared on screen, participants were instructed to select the emotion depicted by the actor by pressing the corresponding button on a response box with buttons labeled as each emotion used in the task. Participants had unlimited time to make a response after presentation of each face stimulus, such that the next face stimulus was displayed on the screen a brief pause after they had responded to the previous image, regardless of labeling accuracy. Participants were given three enforced rest periods during each task, at the end of which the session was resumed. A short break was also offered between completing the two FERT tasks. Upon completion of all experimental procedures, participants were asked to complete a debriefing form and given the opportunity to ask any questions about the study.

### Statistical Analysis

Five repeated measures analyses of variance (ANOVAs) were conducted. For all analyses, the significance level was set at *p* < 0.05. The first ANOVA compared the effects of face set (PAFs, PoFA) and emotion (happy, sad, fear, anger, disgust, surprise and neutral) as within-subjects factors on emotion recognition accuracy scores, controlling for effects of task order and participant gender. The second ANOVA compared effects of face set and emotion as within-subjects factors on emotion misclassifications, defined as the number of times each emotion was selected in error to mislabel another and expressed as a percentage of the total possible number of incorrect responses. As before, we controlled for effects of task order and participant gender. A third ANOVA compared effects of face set, emotion and emotion intensity (10–100%) as within-subjects factors on emotion recognition accuracy scores, controlling for effects of task order and participant gender. Separately for the PAFs and PoFA FERT tasks, we conducted an ANOVA to compare effects of actor gender and emotion as within-subject factors and participant gender as a between-subject factor on emotion recognition accuracy scores. Sad facial expressions were excluded from analysis of the PoFA data, as this included no male actors expressing this emotion. In both analyses, we controlled for effect of task order. Data on actor ethnicity were available for the PAFs but due to the imbalances in both participant and actor ethnicity groups, we could not apply the same ANOVA approach to investigate these interactions. Nevertheless, we presented these data descriptively, as a starting point for future work into the development and implementation of the PAFs. Where *post-hoc* explorations of significant effects and interactions were required, paired samples *t*-tests were used with Bonferroni-corrected significance levels (Supplementary Statistics). Finally, Pearson correlation analyses were used to examine relationships between participant BDI scores, recognition accuracy scores for negatively-valenced emotions (sad, fear, anger, disgust) and recognition accuracy scores for positively or ambiguous -valenced emotions (happy, surprise). We did not control for effects of participant age or years of education as the distribution of these data was skewed toward mostly young, university-educated individuals, and violated assumptions underlying ANOVAs (Table 1). However, due to the sample size and relative homogeneity, it was unlikely that we were powered to detect such effects. Analyses were conducted using IBM SPSS Statistics software (version 25.0).

## Results

### Effect of Face Set and Emotion on Recognition Accuracy

**Figure 1:**
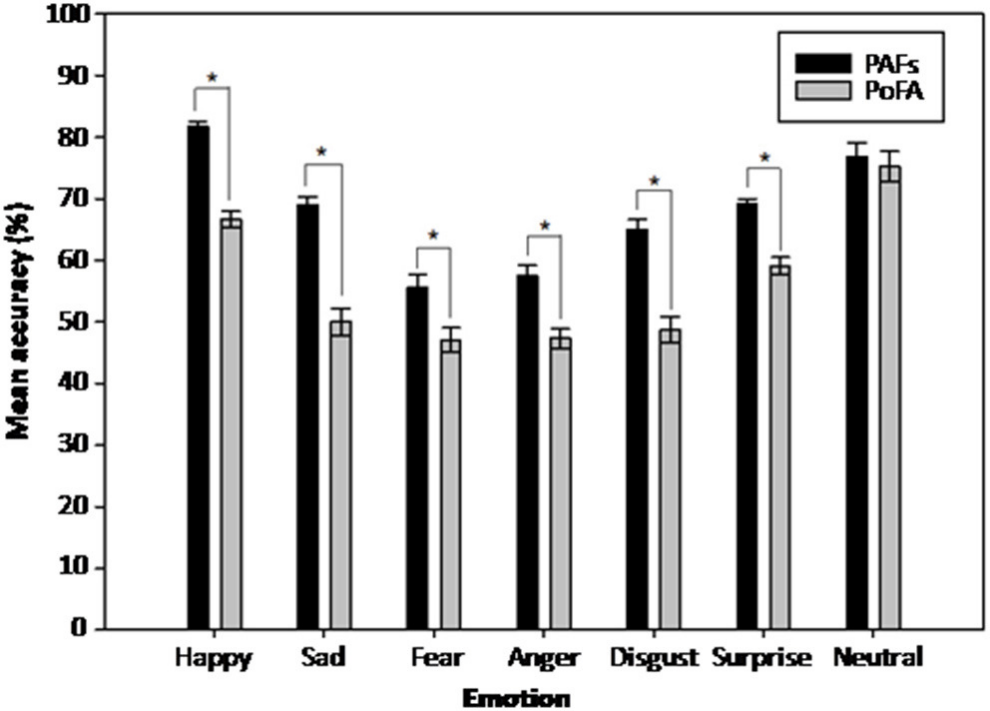
Mean recognition accuracy scores for the two face sets, split by emotion. PAFs, P1vital® Affective Faces set; PoFA, Pictures of Facial Affect. Error bars represent the standard error of the mean. *p < 0.05.

Mauchly's test indicated that the assumption of sphericity had been violated for the within-subjects factor of emotion [χ^2^(20) = 100.27, *p* < 0.001] and the emotion x face set interaction [χ^2^(20) = 62.95, *p* < 0.001]. Therefore, degrees of freedom were corrected using Greenhouse-Geisser estimates of sphericity. The analysis revealed significant main effects of face set [*F*_(1,57)_ = 265.22, *p* < 0.001, ηp^2^ = 0.82], and emotion [*F*_(3.32,189.03)_ = 50.24, *p* < 0.001, ηp^2^ = 0.47]. These main effects were qualified by a significant face set x emotion interaction [*F*_(4.03,229.92)_ = 9.28, *p* < 0.001, ηp^2^ = 0.14]. Further investigation of this interaction using two-tailed paired samples *t*-tests showed that recognition accuracy was significantly greater for all emotions in the PAFs compared to the PoFA, with the exception of neutral faces (Figure 1 and Supplementary Statistics). In spite of differences in overall accuracy, there were consistent response patterns pertaining to both face sets. Notably, out of the six emotions, happiness was the most accurately identified (M = 74.0%, SE = 0.01), and fear was the least accurately identified (M = 51.1%, SE = 0.02) (Figure 1).

### Effect of Face Set and Emotion on Misclassifications

**Figure 2:**
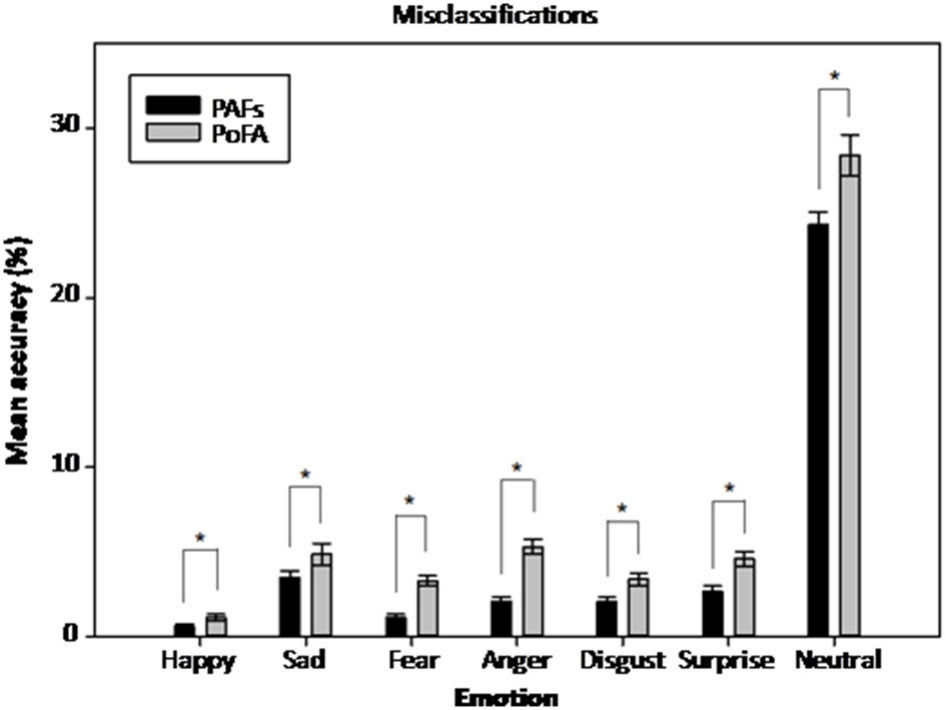
Mean percentage misclassifications for each of the six emotions and neutral expressions for the two face sets. Error bars represent the standard error of the mean. *p < 0.05. PAFs, P1vital® Affective Faces set; PoFA, Pictures of Facial Affect.

As the assumption of sphericity was violated for the within-subjects factor of emotion [χ^2^(20) = 268.57, *p* < 0.001] and the face set x emotion interaction [χ^2^(20) = 177.11, *p* < 0.001], degrees of freedom were corrected using Greenhouse-Geisser estimates of sphericity. As before, there were significant main effects of face set [*F*_(1,57)_ = 279.08, *p* < 0.001, ηp^2^ = 0.83], and emotion [*F*_(1.84,105.11)_ = 361.28, *p* < 0.001, ηp^2^ = 0.86]. On average, more misclassifications were made in FERT tasks employing the PoFA (M = 7.28%, SE = 0.002) compared to the PAFs (M = 5.20%, SE = 0.001). Misclassifications of other emotions as neutral expressions were the most common (MPAFs = 24.3%, SE = 0.01; MPoFA = 28.4%, SE = 0.01) and misclassifications of other emotions as happy were the least common (MPAFs = 0.61%, SE = 0.001; MPoFA = 1.13%, SE = 0.002). This was consistent across both face sets (Figure 2). Furthermore, both face sets showed consistencies regarding common misclassifications for each emotion (Table 2). The two main effects were qualified by a significant face set x emotion interaction [*F*_(2.77,157.91)_ = 5.60, *p* = 0.002, ηp^2^ = 0.09]. The significant interaction indicated variability in the size of the differences in misclassifications between the two face sets, which was lowest for happy misclassifications (MPAFs = 0.61%, SE = 0.001; MPoFA = 1.13%, SE = 0.002) and highest for neutral misclassifications (MPAFs = 24.3%, SE = 0.01; MPoFA = 28.4%, SE = 0.01) (Supplementary Statistics).

**Table 2:** Most frequent misclassifications for each emotion in the two face sets.

**Emotion presented**	**Most common misclassification PAFs**	**Most common misclassification PoFA**
Anger	Disgust (1.19%)	Sad (1.49%), Disgust (1.45%)
Disgust	Anger (1.89%)*	Anger (2.76%)*
Fear	Surprise (2.60%)*	Surprise (3.08%)*
Happy	Sad (0.40%)*	Sad (0.39%)*
Sad	Fear (0.18%)	Fear (1.30%)
Surprise	Fear (0.60%)	Fear (0.94%)

*Significantly more misclassifications compared to misclassifications as other emotions, p < 0.05. PAFs, P1vital® Affective Faces set; PoFA, Pictures of Facial Affect.

### Effects of Emotion Intensity on Recognition Accuracy

**Figure 3:**
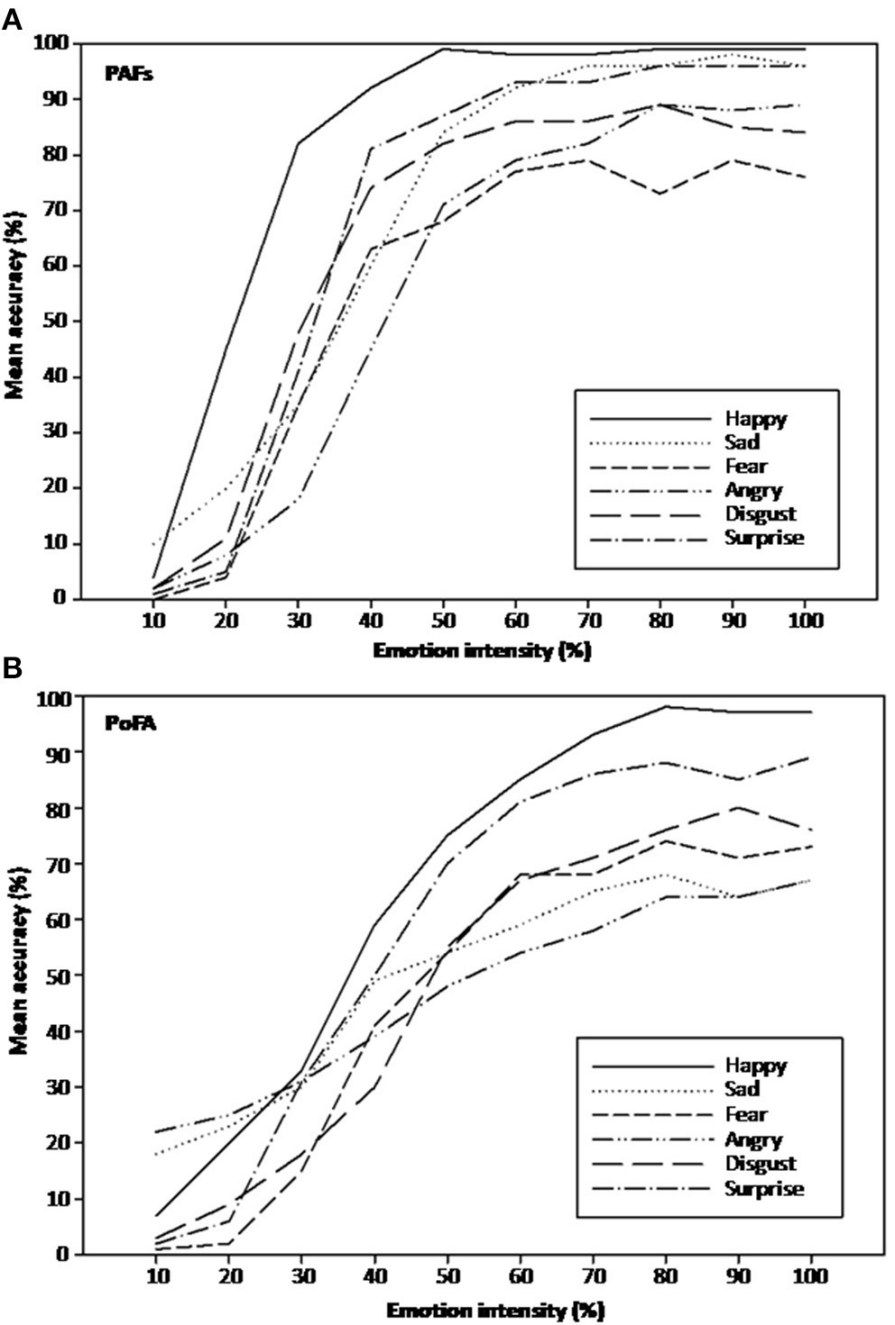
**(A)** Mean recognition accuracy at different emotion intensities for the P1vital®Affective Faces set (PAFs). **(B)** Mean recognition accuracy at different emotion intensities for the Pictures of Facial Affect (PoFA).

The sphericity assumption was violated for the within-subjects factor of emotion intensity [χ^2^(44) = 190.51, *p* < 0.001], face set x emotion intensity [χ^2^(44) = 99.98, *p* < 0.001], emotion x emotion intensity interaction [χ^2^(1034) = 1439.64, *p* < 0.001], and face set x emotion x emotion intensity interaction [χ^2^(1034) = 1406.23, *p* < 0.001]. Correction of the degrees of freedom using Greenhouse-Geisser estimates of sphericity revealed a significant effect of emotion intensity [*F*_(4.53,258.25)_ = 1645.35, *p* < 0.001, ηp^2^ = 0.97], indicating that overall performance improved with increased degree of emotion in the images (Figures 3A,B). There was also a significant face set x intensity interaction [*F*_(6.52,371.77)_ = 48.14, *p* < 0.001, ηp^2^ = 0.46]. Further investigation showed that accuracy to the PAFs was significantly greater compared with the PoFA for all intensities of emotion with the exception of the 10% intensity level [where accuracy to PoFA was significantly greater, *t*(60) = −8.26, *p* < 0.001] and the 20% intensity level (where there was no significant difference between the two face sets). For the PAFs face set, paired *t*-tests revealed significant differences between each successive level of intensity of emotion up to 60%, with the greater intensity levels being more accurately identified. There were no significant differences between the 60–70, 70–80, 80–90, or 90–100% emotion intensity levels, suggestive of a plateau in recognition accuracy (Supplementary Statistics). The PoFA also showed significant differences between each emotion intensity level and the subsequent level up to 80%, with greater intensity levels being more accurately identified. There were no significant differences between the 80–90 or 90–100% emotion intensity levels (Supplementary Statistics).

We also observed a significant emotion x intensity interaction [*F*_(20.75,1182.49)_ = 12.34, *p* < 0.001, ηp^2^ = 0.18]. This was due to some emotions reaching a plateau in recognition accuracy at lower intensities. For example, happy faces from the PAFs were recognized with nearly 100% accuracy at 50% intensity. Accuracy for the other emotions plateaued at higher intensities (Figure 3A). Happy faces in the PoFA reached a plateau in recognition at 80% intensity. The other five emotions did not show this effect, even at the highest intensities (Figure 3B and Supplementary Statistics).

The face set x intensity and emotion x intensity interactions were further qualified by a significant face set x emotion x intensity interaction [*F*_(21.37,1217.82)_ = 18.35, *p* < 0.001, ηp^2^ = 0.24], due to differences in recognition accuracy between the PAFs and the PoFA at different intensities for different emotions. Disgust, fear and happiness showed no difference in accuracy between the two face sets at the lowest (~10–20%) and highest (~70–100%) intensities, although accuracy was significantly higher for the PAFs for moderate intensities (~40–60%). For surprise and sadness, the difference in recognition accuracy in favor of the PAFs became evident at the 40 and 50% intensity levels, respectively, and continued to be significant through to the highest intensity levels. In contrast, recognition accuracy differences between the two face sets for anger were significant at most intensity levels, first in favor of the PoFA (10–30%) and then in favor of the PAFs (50–100%) (Supplementary Statistics).

### Effect of Participant and Actor Demographics on Recognition Accuracy

In both ANOVA analyses, the assumption of sphericity was violated for the emotion x actor gender interaction [PAFs:χ^2^(20) = 81.01, *p* < 0.001; PoFA: χ^2^(14) = 33.97, *p* = 0.002] and the degrees of freedom were corrected as above. For the PAFs, we observed a significant actor gender x emotion interaction [*F*_(3.66,208.68)_ = 31.74, *p* < 0.001, ηp^2^ = 0.36]. Anger [*t*(60) = 7.98, *p* < 0.001, d = 1.02], happiness [*t*(60) = 2.99, *p* = 0.004, d = 0.38] and surprise [*t*(60) = 3.77, *p* < 0.001, d = 0.48] were significantly more accurately identified when depicted by male compared to female actors. Fear [*t*(60) = −3.85, *p* < 0.001, d = 0.49], sadness [*t*(60) = −6.26, *p* < 0.001, d = 0.80] and neutral expressions [*t*(60) = −6.12, *p* < 0.001, d = 0.78] were significantly more accurately identified when depicted by female compared to male actors. The participant gender x actor gender interaction was not significant. For the PoFA, there was a significant effect of actor gender [*F*_(1,57)_ = 46.47, *p* < 0.001, ηp^2^ = 0.45], indicating greater recognition accuracy for emotions displayed by male (M = 57.80, SE = 0.01) compared to female (M = 51.71, SE = 0.01) actors. This was qualified by a significant actor gender x emotion interaction [*F*_(4.03,229.94)_ = 22.00, *p* < 0.001, ηp^2^ = 0.28]. Anger [*t*(60) = 7.57, *p* < 0.001, d = 0.97], disgust [*t*(60) = 7.66, *p* < 0.001, d = 0.98] and fear [*t*(60) = 5.81, *p* < 0.001, d = 0.74] were significantly more accurately identified when displayed by male compared to female actors. In contrast, surprise [*t*(60) = 6.42, *p* < 0.001, d = 0.82] was significantly more accurately identified when displayed by female compared to male actors. Finally, we observed a significant effect of subject gender [*F*_(1,57)_ = 4.23, *p* = 0.04, ηp^2^ = 0.07], indicating that female participants (M = 55.57%, SE = 0.06) were more accurate than male participants (M = 51.97%, SE = 0.10) at recognizing expressions from the PoFA. The participant gender x actor gender interaction was not significant (Table 3).

**Table 3:** Mean recognition accuracy scores for each emotion and actor gender in the two face sets.

**Emotion**	**PAFs, female**	**PAFs, male**	**PoFA, female**	**PoFA, male**
Happy	80.7%, SE = 0.01	84.1%, SE = 0.01	66.2%, SE = 0.02	67.1%, SE = 0.02
Fear	57.4%, SE = 0.02	49.5%, SE = 0.02	43.3%, SE = 0.02	57.1%, SE = 0.03
Anger	53.6%, SE = 0.02	67.9%, SE = 0.02	39.3%, SE = 0.02	54.9%, SE = 0.02
Disgust	64.0%, SE = 0.02	66.9%, SE = 0.02	39.8%, SE = 0.03	57.1%, SE = 0.02
Surprise	67.7%, SE = 0.01	72.5%, SE = 0.01	64.2%, SE = 0.01	53.4%, SE = 0.02
Neutral	83.1%, SE = 0.03	62.3%, SE = 0.03	74.0%, SE = 0.03	77.1%, SE = 0.03

*PAFs, P1vital® Affective Faces set; PoFA, Pictures of Facial Affect; SE, standard error of the mean*.

Mean recognition accuracy scores for the PAFs for each participant and actor ethnicity are displayed in Table 4. We reduced the comparison in terms of actor ethnicity to only two levels (White and Black) because the other two ethnic categories (Asian and Mixed) were represented by one actor only. While no specific conclusions can be drawn from these data, they appear to suggest that some emotions were better recognized when expressed by actors of the same ethnicity, and others were better recognized when expressed by actors of a different ethnicity.

**Table 4:** Mean recognition accuracy scores for the P1vital®Affective Faces set (PAFs) for each emotion, participant ethnicity and actor ethnicity.

	**Participant ethnicity**							
	**White**				**Black**			
**Emotion**	**Actor ethnicity**				**Actor ethnicity**			
	**White**		**Black**		**White**		**Black**	
Happy	83.5%	SE = 0.01	81.0%	SE = 0.01	85.6%	SE = 0.02	76.7%	SE = 0.02
Fear	50.8%	SE = 0.02	59.3%	SE = 0.04	46.7%	SE = 0.07	51.1%	SE = 0.07
Anger	70.5%	SE = 0.02	64.0%	SE = 0.02	61.1%	SE = 0.07	62.2%	SE = 0.04
Disgust	68.3%	SE = 0.02	60.8%	SE = 0.03	65.6%	SE = 0.06	65.6%	SE = 0.04
Surprise	72.3%	SE = 0.02	74.5%	SE = 0.02	71.1%	SE = 0.03	72.2%	SE = 0.04
Neutral	68.3%	SE = 0.03	87.5%	SE = 0.04	77.8%	SE = 0.09	94.4%	SE = 0.06

*SE, standard error of the mean*.

### Relationship Between BDI Scores and Recognition Accuracy of Positive and Negative Emotional Faces

The relationship between BDI score and recognition accuracy for positive (happy, surprise) or negative (sad, fear, anger, disgust) emotional facial expressions across both FERT tasks did not reach significance in either case.

## Discussion

Overall, the current results contribute to the initial validation of the PAFs as a contemporary face set that can be used in studies of emotional processing. Participants were more accurate in identifying emotions from the PAFs compared to the PoFA, although there were consistencies in terms of participant performance between the two face sets in terms of both accurate and inaccurate patterns of responding. Happy expressions were the most accurately identified, while fearful expressions were the least accurately identified. When responses were inaccurate, participants were most likely to misclassify emotions from both face sets as neutral.

Our comparisons of emotion recognition accuracy between the PAFs and PoFA face sets revealed a significant difference in performance, where recognition of all emotions was greater for the PAFs at intensities of 30% and greater. This suggests that the emotional stimuli in the PAFs face set are more easily identifiable than those in the PoFA. We also observed a significant effect of emotion intensity, where accuracy improved as the degree of emotion shown in the facial stimuli increased. Significant improvement in recognition accuracy was identified between all 10% increases in emotion intensity up to 60 and 80% for the PAFs and PoFA, respectively. This indicates that both the PAFs and the PoFA faces reached a plateau in performance accuracy, where further increases in emotion intensity no longer impacted participant ability to identify faces. Although performance accuracy in the PAFs passed the 90% mark for some emotions at the highest intensities, the face set does not seem to be at risk of suffering from ceiling effects when compared to the PoFA. This is partly due to the inherent difficulty of recognizing low intensity emotional expressions, as evidenced by lack of a difference in recognition accuracy between the two face sets at low emotion intensities, and the relative ease with which high intensity expressions can be identified. For half of the emotions employed in this study, we did not observe a difference in recognition accuracy between the two face sets at intensity levels of 70% and above. Therefore, the PAFs should be similarly sensitive to manipulations of subject emotional recognition abilities, such as the use of drugs that alter processing of emotional information.

There was also a significant main effect of emotion, with participants showing greater accuracy for some emotions than others. This was qualified by an interaction between face set and emotion, which indicated that recognition accuracy scores were significantly greater for all emotions in the PAFs compared to the PoFA, with the exception of neutral faces. This may be due to the similar levels of ambiguity in black and white and color pictures of unemotional facial stimuli. Furthermore, the significant face set, emotion and intensity interaction clarified that these differences were apparent at some but not all intensity levels for most emotions.

While performance accuracy differed quantitatively between the two face sets, there were qualitative similarities in performance with happy expressions being more accurately identified and less likely to be misclassified as other facial expressions of emotion. Additionally, accuracy of recognition of fearful faces was lower than that of other emotions for both face sets. The idea that fear may be more difficult to identify than other primary emotions has been previously described (23), and our results suggest that the effect appears to be independent of the face set used.

Other consistencies between the two face sets were apparent in the pattern of incorrect responses by our study participants. Although the overall percentage of misclassifications was lower for the PAFs compared to the PoFA, the types of misclassifications made were nearly identical in the two face sets. Participants were most likely to misclassify other emotions as neutral and least likely to misclassify them as happy. Anger was most often misclassified as disgust (additionally as sad in the PoFA), disgust as anger, fear as surprise, sad as fear, surprise as fear, and happy as sad in both face sets. Such consistent patterns of response between the PAF and the PoFA strengthen the idea that these tasks can provide reliable biomarkers, and support the use of the PAFs as an alternative face set to the PoFA.

Our investigation of the effect of participant and actor demographics showed no significant interactions between participant gender and actor gender. Female participants were significantly better than male participants at recognizing emotions, but only when classifying faces from the PoFA. We also observed significant interactions between emotion and actor gender for both face sets. In both the PAFs and the PoFA, anger was best recognized when displayed by male actors rather than female actors, a result consistent with previous literature (24). Besides this commonality however, positive emotions (happiness, surprise) were better recognized when displayed by male actors in the PAFs, and female actors in the PoFA. The opposite was true for negative emotions (fear, disgust), which were better recognized when displayed by female actors in the PAFs and male actors in the PoFA. Nevertheless, only one male actor was used to display happiness, sadness, fear, anger, disgust and surprise in the PAFs. Thus, any significant results may have been influenced by the individual characteristics of this particular actor which were unrelated to gender. In the future, better gender balancing of the emotional stimuli in the PAFs should lead to increased clarity and more accurate exploration of the participant x actor gender interaction, since gender may make faces inherently resemble certain emotions (25).

Contrary to expectation, our descriptive analysis of the interaction between participant and actor ethnicity did not reveal a significant own-race effect. Reported means suggest that some emotions were better recognized when expressed by actors of the same ethnicity, and others were better recognized when expressed by actors of a different ethnicity. Because the PoFA only includes Caucasian actors, this result does not substantiate our finding of equivalence between the two face sets. However, it does highlight the importance of building an ethnically diverse face set for use in tests of facial emotion recognition, as this adds to its ability to convey the designated emotions to ethnically diverse participant samples. In future, our findings should be substantiated by using a FERT task employing equal numbers of actors of varying ethnicities. At present, our PAFs FERT included only two black, one Asian and one mixed ethnicity actor; our participant sample was also predominantly white, limiting the conclusions that can be drawn.

Lastly, we hypothesized that there will be a relationship between BDI scores and recognition accuracy for both positively- and negatively-valenced emotional faces. Previous research has consistently linked low mood to decreased accuracy in identifying and remembering positive information (9, 26). However, in the current study, the relationships between BDI scores and recognition accuracy for positively- or negatively-valenced faces did not reach statistical significance. This is most likely attributable to the limited range of BDI scores in our sample (1–24 out of a total possible of 61) and the fact that most participants could not be classified as dysphoric or depressed (78% had BDI scores between 0 and 10, which are interpreted as “normal mood fluctuations”). The relationship between mood and emotion recognition is likely to remain of interest as research into its link with antidepressant function continues to expand. Therefore, opportunities of employing the FERT task in clinical populations, together with well-validated clinical tools that measure severity of depressive symptoms, are likely to be plentiful in the future.

There are a number of limitations that should be noted for this study. Firstly, we recruited a total of 61 participants, a sample size slightly lower than that reported in similar studies aiming to validate novel face sets (3, 13). As previously mentioned, the sample size and ethnic diversity of actors in the PAFs was also limited. Nevertheless, this size was sufficiently large to produce statistically sound results, with many of our observed significant effects having medium or large effect sizes. Secondly, 92% of the participants were 18–29 years of age. Only five participants were aged between 30 and 39 years, and only three were 40–49 years-old. While this participant age distribution is not uncommon in validation studies of other face sets (13, 14), it meant we were unable to control for participant age in our analyses or investigate the effects of age on emotion recognition in the two face sets. Further studies of the PAFs should include a more representative sample of participants aged 18–75 years, in order to validate their use with older adults and extend their applicability. Previous studies have found that age has the greatest impact on the recognition of the sad emotion and that the greatest age effect is detectable at the 50% level of intensity (27). Furthermore, age differences in emotion recognition may also be task/context-specific in addition to emotion-specific (28). This highlights the importance of including older subjects in validation studies which compare participant performance on tasks employing novel face sets with their performance on tasks employing well-established facial stimuli.

Many studies have consistently shown that the emotional content, or type of emotion expressed, can affect the accuracy with which emotional facial expressions are recognized (29–31). It is suggested that we are “hard-wired” to recognize threats, such as that conveyed by a fearful or angry facial expression, and this receives some support from studies that demonstrate that the detection of angry facial expressions is more accurate than happy expressions (32). However, the apparent biological advantage (implicit recognition) that humans have in detecting threat is not apparent when subjects are asked explicitly to identify facial emotions. As we found in this study, happiness is the most accurately recognized facial emotion (29–31) and fear is often the least accurately recognized (29). Wells et al. suggest that different expressions may vary with respect to their function. For example, happy smiling faces are primarily an explicit social communication, whereas the threat implicit in anger may be a cue for danger in the environment. Such danger may be recognized in the absence of explicit awareness or identification (23, 33). It may be that the performance advantage observed with the PAF occurs due to the high-definition color photographs which display the emotion more intensely than that conveyed by the black and white, lower definition black and white photos of the PoAF set. Further studies may be able to elucidate whether the performance advantage seen with the PAF is due to the amount of emotion or the degree of intensity of the facial expressions.

In conclusion, we found a similar pattern of participant performance on two FERT tasks employing the PAFs and the PoFA (12), with emotion recognition accuracy scores being marginally higher for the PAFs. This result suggests that the PAFs can be reliably deployed to measure facial emotion recognition in contemporary research. Having a modern, ethnically diverse alternative to the PoFA is important for a number of basic research and clinical research applications, for example measuring drug or other intervention-induced changes in affective processing in both healthy individuals and psychopathology. As an example, it has been successfully deployed in a recent large-scale clinical trial with more than 900 patients recruited from primary care centers across Europe. As the faces data set is large and diverse, several novel face sets were derived that enabled the FERT be deployed on successive weekly trials in which treatment was modified and a patient's negative bias in response to that change was assessed. The high-resolution images facilitated the deployment of FERT on PCs, laptops, tables and smart phones enabling patients to use their own devices to complete the task at home (34).

## Data Availability Statement

The original contributions presented in the study are included in the article/Supplementary Material, further inquiries can be directed to the corresponding author/s.

## Ethics Statement

The studies involving human participants were reviewed and approved by Ethical Committee of Brunel University and Ethical Committee of University of Birmingham. The patients/participants provided their written informed consent to participate in this study.

## Author Contributions

JR, LV, and GD constructed the PAFs, carried out the study, analyzed the data, and wrote the manuscript. JK constructed the PAFs and carried out the study. JT carried out the study, analyzed the data, and wrote the manuscript. AR analyzed the data and wrote the manuscript. CD and SH wrote the manuscript. All authors contributed to the article and approved the submitted version.

## Funding

This work was supported by P1vital Products Ltd.

## Conflict of Interest

GD was a full-time employee of P1vital Ltd. AR, JR, CD, and LV were employed by P1vital Ltd. at the time this research was conducted. JK was a full-time employee of P1vital Products Ltd. The P1vital Affective Faces are the property of P1vital Products Ltd. GD and CD are co-owners and major shareholders of P1vital Ltd. GD, JK, and CD are major shareholders in P1vital Products Ltd. The remaining authors declare that the research was conducted in the absence of any commercial or financial relationships that could be construed as a potential conflict of interest.

## Publisher's Note

All claims expressed in this article are solely those of the authors and do not necessarily represent those of their affiliated organizations, or those of the publisher, the editors and the reviewers. Any product that may be evaluated in this article, or claim that may be made by its manufacturer, is not guaranteed or endorsed by the publisher.
