# Supplementary material for: Validation of the P1vital® Faces Set for Use as Stimuli in Tests of Facial Emotion Recognition
Source: Front Psychiatry. 2022 Feb 11;13:663763. doi: 10.3389/fpsyt.2022.663763 (PMC8874121; doi:10.3389/fpsyt.2022.663763)
Supplement: Supplementary file 1 [file Data_Sheet_1.pdf]

Supplementary statistics

Table 1.

Comparison of mean recognition accuracy scores for the six different emotions and neutral expressions between the two face sets.

**Emotion**	**Mean recognition accuracy (%) PAFs**	**Mean recognition accuracy (%) PoFA**	**Paired t-test result (PAFs vs PoFA)**
**Happy**	81.52%, SE = 0.01	66.43%, SE = 0.01	t(60) = 15.1, p < .001, d = 1.93*
**Sad**	68.73%, SE = 0.01	49.75%, SE = 0.02	t(60) = 10.7, p < .001, d = 1.37*
**Fear**	55.45%, SE = 0.02	46.76%, SE = 0.02	t(60) = 4.52, p < .001, d = 0.58*
**Anger**	57.17%, SE = 0.02	47.09%, SE = 0.02	t(60) = 7.26, p < .001, d = 0.93*
**Disgust**	64.71%, SE = 0.02	48.40%, SE = 0.02	t(60) = 8.20, p < .001, d = 1.05*
**Surprise**	68.89%, SE = 0.01	58.77%, SE = 0.01	t(60) = 7.56, p < .001, d = 0.97*
**Neutral**	76.89%, SE = 0.02	75.25%, SE = 0.02	t(60) = 0.61, p = .543

Note: PAFs = P1vital® Affective Faces set. PoFA = Pictures of Facial Affect. SE = standard error of the mean. *p<0.007 (Bonferroni corrected).

Table 2.

Comparison of percentage misclassifications for the six different emotions and neutral expressions between the two face sets.

**Emotion**	**Misclassifications (%) PAFs**	**Misclassifications (%) PoFA**	**Paired t-test result (PAFs vs PoFA)**
**Happy**	0.61%, SE = 0.001	1.31%, SE = 0.002	t(60) = -3.18, p = .002, d = 0.41*
**Sad**	3.50%, SE = 0.004	4.86%, SE = 0.01	t(60) = -2.82, p = .006, d = 0.36*
**Fear**	1.14%, SE = 0.002	3.29%, SE = 0.003	t(60) = -7.67, p < .001, d = 0.98*
**Anger**	2.05%, SE = 0.003	5.31%, SE = 0.01	t(60) = -6.71, p < .001, d = 0.86*
**Disgust**	2.09%, SE = 0.002	3.37%, SE = 0.004	t(60) = -4.00, p < .001, d = 0.51*
**Surprise**	2.66%, SE = 0.003	4.59%, SE = 0.004	t(60) = -4.56, p < .001, d = 0.58*
**Neutral**	24.34%, SE = 0.01	28.42%, SE = 0.01	t(60) = -4.97, p < .001, d = 0.64*

Note: PAFs = P1vital® Affective Faces set. PoFA = Pictures of Facial Affect. SE = standard error of the mean. *p<0.007 (Bonferroni corrected).

Table 3

Comparison of mean recognition accuracy scores between each successive intensity level across all emotions for the PAFs.

Intensity pair	Mean recognition accuracy (%) for lower intensity	Mean recognition accuracy (%) for higher intensity	Paired t-test result (lower intensity vs higher intensity)
**10 vs 20%**	3.07%, SE = 0.01	15.51%, SE = 0.01	t(60) = -12.43, p < .001, d = 1.59*
**20 vs 30%**	15.51%, SE = 0.01	43.17%, SE = 0.02	t(60) = -16.94, p < .001, d = 2.17*
**30 vs 40%**	43.17%, SE = 0.02	69.26%, SE = 0.02	t(60) = -17.15, p < .001, d = 2.20*
**40 vs 50%**	69.26%, SE = 0.02	81.83%, SE = 0.02	t(60) = -9.45, p < .001, d = 1.21*
**50 vs 60%**	81.83%, SE = 0.02	87.77%, SE = 0.01	t(60) = -5.81, p < .001, d = 0.74*
**60 vs 70%**	87.77%, SE = 0.01	89.00%, SE = 0.01	t(60) = -1.15, p = .254
**70 vs 80%**	89.00%, SE = 0.01	90.23%, SE = 0.01	t(60) = -1.43, p = .159
**80 vs 90%**	90.23%, SE = 0.01	90.85%, SE = 0.01	t(60) = -0.72, p = .477
**90 vs 100%**	90.85%, SE = 0.01	90.10%, SE = 0.01	t(60) = 0.76, p = .435

Note: PAFs = P1vital® Affective Faces set. PoFA = Pictures of Facial Affect. SE = standard error of the mean. *p<0.006 (Bonferroni corrected).

Table 4

Comparison of mean recognition accuracy scores between each successive intensity level across all emotions for the PoFA.

Intensity pair	Mean recognition accuracy (%) for lower intensity	Mean recognition accuracy (%) for higher intensity	Paired t-test result (lower intensity vs higher intensity)
**10 vs 20%**	9.02%, SE = 0.01	14.00%, SE = 0.01	t(60) = -5.37, p < .001, d = 0.69*
**20 vs 30%**	14.00%, SE = 0.01	26.30%, SE = 0.01	t(60) = -10.97, p < .001, d = 1.41*
**30 vs 40%**	26.30%, SE = 0.01	44.60%, SE = 0.02	t(60) = -14.75, p < .001, d = 1.89*
**40 vs 50%**	44.60%, SE = 0.02	59.29%, SE = 0.02	t(60) = -11.54, p < .001, d = 1.48*
**50 vs 60%**	59.29%, SE = 0.02	69.13%, SE = 0.02	t(60) = -6.89, p < .001, d = 0.88*
**60 vs 70%**	69.13%, SE = 0.02	73.63%, SE = 0.02	t(60) = -3.98, p < .001, d = 0.51*
**70 vs 80%**	73.63%, SE = 0.02	78.01%, SE = 0.01	t(60) = -3.43, p = .001, d = 0.44*
**80 vs 90%**	78.01%, SE = 0.01	76.64%, SE = 0.01	t(60) = 1.32, p = .191
**90 vs 100%**	76.64%, SE = 0.01	78.07%, SE = 0.02	t(60) = -1.18, p = .243

Note: PAFs = P1vital® Affective Faces set. PoFA = Pictures of Facial Affect. SE = standard error of the mean. *p<0.006 (Bonferroni corrected).

Table 5

Comparison of mean recognition accuracy scores for each emotion intensity level between the two face sets.

Intensity	Mean recognition accuracy (%) PAFs	Mean recognition accuracy (%) PoFA	Paired t-test result (PAFs vs PoFA)
**10%**	3.07%, SE = 0.01	9.02%, SE = 0.01	t(60) = -8.27, p < .001, d = 1.06*
**20%**	15.51%, SE = 0.01	14.00%, SE = 0.01	t(60) = 1.30, p = .199
**30%**	43.17%, SE = 0.02	26.30%, SE = 0.01	t(60) = 9.56, p < .001, d = 1.22*
**40%**	69.26%, SE = 0.02	44.60%, SE = 0.02	t(60) = 15.81, p < .001, d = 2.03*
**50%**	81.83%, SE = 0.02	59.29%, SE = 0.02	t(60) = 12.83, p < .001, d = 1.64*
**60%**	87.77%, SE = 0.01	69.13%, SE = 0.02	t(60) = 14.33, p < .001, d = 1.83*
**70%**	89.00%, SE = 0.01	73.63%, SE = 0.02	t(60) = 8.68, p < .001, d = 1.11*
**80%**	90.23%, SE = 0.01	78.01%, SE = 0.01	t(60) = 8.97, p < .001, d = 1.15*
**90%**	90.85%, SE = 0.01	76.64%, SE = 0.01	t(60) = 9.96, p < .001, d = 1.28*
**100%**	90.10%, SE = 0.01	78.07%, SE = 0.02	t(60) = 8.19, p < .001, d = 1.05*

Note: PAFs = P1vital® Affective Faces set. PoFA = Pictures of Facial Affect. SE = standard error of the mean. *p<0.005 (Bonferroni corrected).

Table 6

Comparison of mean recognition accuracy scores for **anger** at each intensity level between the two face sets.

Intensity	Mean recognition accuracy (%) PAFs	Mean recognition accuracy (%) PoFA	Paired t-test result (PAFs vs PoFA)
**10%**	1.64%, SE = 0.001	22.13%, SE = 0.03	t(60) = -7.94, p < .001, d = 1.02*
**20%**	7.79%, SE = 0.02	24.59%, SE = 0.03	t(60) = -5.67, p < .001, d = 0.73*
**30%**	18.03%, SE = 0.03	31.15%, SE = 0.03	t(60) = -4.52, p < .001, d = 0.58*
**40%**	45.49%, SE = 0.04	38.52%, SE = 0.03	t(60) = 1.78, p = .081
**50%**	71.31%, SE = 0.03	47.54%, SE = 0.03	t(60) = 7.37, p < .001, d = 0.94*
**60%**	79.10%, SE = 0.03	54.10%, SE = 0.03	t(60) = 8.39, p < .001, d = 1.07*

**70%**	82.38%, SE = 0.03	57.79%, SE = 0.02	t(60) = 8.33, p < .001, d= 1.07*
**80%**	88.52%, SE = 0.02	63.93%, SE = 0.02	t(60) = 7.88, p < .001, d= 1.01*
**90%**	88.11%, SE = 0.02	63.93%, SE = 0.02	t(60) = 8.83, p < .001, d = 1.13*
**100%**	89.34%, SE = 0.02	67.21%, SE = 0.02	t(60) = 7.56, p < .001, d = 0.97*

Note: PAFs = P1vital® Affective Faces set. PoFA = Pictures of Facial Affect. SE = standard error of the mean. *p<0.005 (Bonferroni corrected).

Table 7

Comparison of mean recognition accuracy scores for **disgust** at each intensity level between the two face sets.

**Intensity**	**Mean recognition accuracy (%) PAFs**	**Mean recognition accuracy (%) PoFA**	**Paired t-test result (PAFs vs PoFA)**
**10%**	2.05%, SE = 0.01	2.87%, SE = 0.01	t(60) = -0.57, p = .568
**20%**	11.07%, SE = 0.02	9.02%, SE = 0.02	t(60) = 0.78, p = .439
**30%**	48.36%, SE = 0.04	18.03%, SE = 0.02	t(60) = 7.12, p < .001, d = 0.91*
**40%**	73.77%, SE = 0.04	29.51%, SE = 0.03	t(60) = 10. 67, p < .001, d = 1.37*
**50%**	81.97%, SE = 0.03	54.51%, SE = 0.03	t(60) = 6.56, p < .001, d = 0.85*
**60%**	86.48%, SE = 0.02	67.21%, SE = 0.04	t(60) = 4.89, p < .001, d = 0.63*
**70%**	86.07%, SE = 0.02	71.31%, SE = 0.03	t(60) = 3.93, p < .001, d = 0.50*
**80%**	88.52%, SE = 0.02	76.23%, SE = 0.03	t(60) = 3.39, p = .001, d = 0.43*
**90%**	84.84%, SE = 0.03	79.51%, SE = 0.03	t(60) = 1.24, p = .220
**100%**	84.02%, SE = 0.03	75.82%, SE = 0.04	t(60) = 2.03, p = .047

Note: PAFs = P1vital® Affective Faces set. PoFA = Pictures of Facial Affect. SE = standard error of the mean. *p<0.005 (Bonferroni corrected).

Table 8

Comparison of mean recognition accuracy scores for **fear** at each intensity level between the two face sets.

**Intensity**	**Mean recognition accuracy (%) PAFs**	**Mean recognition accuracy (%) PoFA**	**Paired t-test result (PAFs vs PoFA)**
**10%**	0.00%, SE = 0.00	0.82%, SE = 0.01	t(60) = -1.43, p = .159

**20%**	4.10%, SE = 0.01	1.64%, SE = 0.01	t(60) = 1.76, p = .083
**30%**	34.84, SE = 0.03	15.16%, SE = 0.02	t(60) = 4.85, p < .001, d = 0.62*
**40%**	63.10%, SE = 0.03	41.39%, SE = 0.03	t(60) = 5.77, p < .001, d = 0.74*
**50%**	67.62%, SE = 0.04	54.10%, SE = 0.03	t(60) = 3.19, p = .002, d = 0.41*
**60%**	77.46%, SE = 0.03	68.03%, SE = 0.04	t(60) = 2.80, p = .007
**70%**	78.69%, SE = 0.03	68.03%, SE = 0.04	t(60) = 2.32, p = .023
**80%**	73.36%, SE = 0.04	74.18%, SE = 0.03	t(60) = -0.21, p = .835
**90%**	79.10%, SE = 0.03	70.90%, SE = 0.03	t(60) = 2.05, p = 0.45
**100%**	76.23%, SE = 0.04	73.36%, SE = 0.04	t(60) = 0.77, p = .447

Note: PAFs = P1vital® Affective Faces set. PoFA = Pictures of Facial Affect. SE = standard error of the mean. *p<0.005 (Bonferroni corrected).

Table 9

Comparison of mean recognition accuracy scores for **happy** at each intensity level between the two face sets.

**Intensity**	**Mean recognition accuracy (%) PAFs**	**Mean recognition accuracy (%) PoFA**	**Paired t-test result (PAFs vs PoFA)**
**10%**	4.10%, SE = 0.01	7.38%, SE = 0.02	t(60) = -1.66, p = .103
**20%**	45.49%, SE = 0.03	20.08%, SE = 0.03	t(60) = 7.51, p < .001, d = 0.96*
**30%**	81.97%, SE = 0.03	32.79%, SE = 0.03	t(60) = 14.23, p < .001, d = 1.82*
**40%**	92.21%, SE = 0.02	58.61%, SE = 0.03	t(60) = 11.09, p < .001, d = 1.42*
**50%**	98.77%, SE = 0.01	75.41%, SE = 0.03	t(60) = 8.97, p < .001, d = 1.15*
**60%**	97.95%, SE = 0.01	85.25%, SE = 0.02	t(60) = 5.69, p < .001, d = 0.73*
**70%**	97.95%, SE = 0.01	93.44%, SE = 0.02	t(60) = 3.02, p = .004, d = 0.39*
**80%**	99.18%, SE = 0.01	97.95%, SE = 0.01	t(60) = 1.14, p = .260
**90%**	98.77%, SE = 0.01	96.72%, SE = 0.01	t(60) = 1.52, p = .133
**100%**	98.77%, SE = 0.01	96.72%, SE = 0.01	t(60) = 1.69, p = .096

Note: PAFs = P1vital® Affective Faces set. PoFA = Pictures of Facial Affect. SE = standard error of the mean. *p<0.005 (Bonferroni corrected).

Table 10

Comparison of mean recognition accuracy scores for **sad** at each intensity level between the two face sets.

Intensity	Mean recognition accuracy (%) PAFs	Mean recognition accuracy (%) PoFA	Paired t-test result (PAFs vs PoFA)
**10%**	9.84%, SE = 0.02	18.44%, SE = 0.03	t(60) = -3.08, p = .003, d = 0.39*
**20%**	19.67%, SE = 0.02	22.95%, SE = 0.03	t(60) = - 0.96, p = .343
**30%**	35.25%, SE = 0.03	29.92%, SE = 0.03	t(60) = 1.44, p = .155
**40%**	59.84%, SE = 0.03	49.18%, SE = 0.03	t(60) = 2.65, p = .010
**50%**	84.02%, SE = 0.03	54.10%, SE = 0.03	t(60) = 8.44, p < .001, d = 1.08*
**60%**	92.21%, SE = 0.02	59.10%, SE = 0.04	t(60) = 9.50, p < .001, d = 1.22*
**70%**	95.90%, SE = 0.01	65.16%, SE = 0.03	t(60) = 9.39, p < .001, d = 1.20*
**80%**	95.90%, SE = 0.01	68.03%, SE = 0.03	t(60) = 9.34, p < .001, d = 1.20*
**90%**	98.36%, SE = 0.01	63.93%, SE = 0.04	t(60) = 9.66, p < .001, d = 1.24*
**100%**	96.31%, SE = 0.01	66.80%, SE = 0.04	t(60) = 7.93, p < .001, d = 1.02*

Note: PAFs = P1vital® Affective Faces set. PoFA = Pictures of Facial Affect. SE = standard error of the mean. *p<0.005 (Bonferroni corrected).

Table 11

Comparison of mean recognition accuracy scores for **surprise** at each intensity level between the two face sets.

Intensity	Mean recognition accuracy (%) PAFs	Mean recognition accuracy (%) PoFA	Paired t-test result (PAFs vs PoFA)
**10%**	0.82%, SE = 0.01	2.46%, SE = 0.01	t(60) = -1.43, p = .159
**20%**	4.92%, SE = 0.01	5.74%, SE = 0.02	t(60) = -0.35, p = .727
**30%**	40.57%, SE = 0.03	30.74%, SE = 0.02	t(60) = 2.69, p = .009
**40%**	81.15%, SE = 0.03	50.41%, SE = 0.03	t(60) = 7.56, p < .001, d = 0.97*
**50%**	87.30%, SE = 0.02	70.08%, SE = 0.03	t(60) = 5.09, p < .001, d = 0.65*
**60%**	93.44%, SE = 0.02	81.15%, SE = 0.03	t(60) = 4.52, p < .001, d = 0.58*
**70%**	93.03%, SE = 0.01	86.07%, SE = 0.03	t(60) = 2.14, p = .037
**80%**	95.90%, SE = 0.01	87.70%, SE = 0.02	t(60) = 3.43, p = 001, d = 0.44*

**90%**	95.90%, SE = 0.01	84.84%, SE = 0.03	t(60) = 3.27, p = .002, d = 0.42*
**100%**	95.90%, SE = 0.01	88.52%, SE = 0.03	t(60) = 2.51, p = .015

Note: PAFs = P1vital® Affective Faces set. PoFA = Pictures of Facial Affect. SE = standard error of the mean. *p<0.005 (Bonferroni corrected).

Table 13.

Means and standard errors for each emotion at each intensity level, for the two face sets.

					PAFs					
Emotion					Intensity					
	**10%**	**20%**	**30%**	**40%**	**50%**	**60%**	**70%**	**80%**	**90%**	**100%**
Happy	4.10%, SE = 0.01	45.49%, SE = 0.03	81.97%, SE = 0.03	92.21%, SE = 0.02	98.77%, SE = 0.01	97.95%, SE = 0.01	97.95%, SE = 0.01	99.18%, SE = 0.01	98.77%, SE = 0.01	98.77%, SE = 0.01
Sad	9.84%, SE = 0.02	19.67%, SE = 0.02	35.25%, SE = 0.03	59.84%, SE = 0.03	84.02%, SE = 0.03	92.21%, SE = 0.02	95.90%, SE = 0.01	95.90%, SE = 0.01	98.36%, SE = 0.01	96.31%, SE = 0.01
Fear	0.00%, SE = 0.00	4.10%, SE = 0.01	34.04%, SE = 0.03	63.10%, SE = 0.03	67.62%, SE = 0.04	77.46%, SE = 0.03	78.69%, SE = 0.03	73.36%, SE = 0.04	79.10%, SE = 0.03	76.23%, SE = 0.04
Anger	1.64%, SE = 0.01	7.79%, SE = 0.02	18.03%, SE = 0.03	45.49%, SE = 0.04	71.31%, SE = 0.03	79.10%, SE = 0.03	82.38%, SE = 0.03	88.52%, SE = 0.02	88.11%, SE = 0.02	89.34%, SE = 0.02
Disgust	2.05%, SE = 0.01	11.07%, SE = 0.02	48.36%, SE = 0.04	73.77%, SE = 0.04	81.97%, SE = 0.03	86.48%, SE = 0.02	86.07%, SE = 0.02	88.52%, SE = 0.02	84.84%, SE = 0.03	84.02%, SE = 0.03
Surprise	0.82%, SE = 0.01	4.92%, SE = 0.01	40.57%, SE = 0.03	81.15%, SE = 0.03	87.30%, SE = 0.02	93.44%, SE = 0.02	93.03%, SE = 0.02	95.90%, SE = 0.01	95.90%, SE = 0.01	95.90%, SE = 0.01
Average	3.07%, SE = 0.04	15.51%, SE = 0.08	43.17%, SE = 0.02	69.26%, SE = 0.02	81.83%, SE = 0.02	87.77%, SE = 0.01	89.00%, SE = 0.01	90.23%, SE = 0.01	90.85%, SE = 0.01	90.10%, SE = 0.01
					**PoFA**					
Emotion					**Intensity**					
	**10%**	**20%**	**30%**	**40%**	**50%**	**60%**	**70%**	**80%**	**90%**	**100%**
Happy	7.38%, SE = 0.02	20.08%, SE = 0.03	32.79%, SE = 0.03	58.61%, SE = 0.03	75.41%, SE = 0.03	85.25%, SE = 0.02	93.44%, SE = 0.02	97.95%, SE = 0.01	96.72%, SE = 0.01	96.72%, SE = 0.01
Sad	18.44%, SE = 0.03	22.95%, SE = 0.03	29.92%, SE = 0.03	49.18%, SE = 0.03	54.10%, SE = 0.03	59.02%, SE = 0.04	65.16%, SE = 0.03	68.03%, SE = 0.03	63.93%, SE = 0.04	66.80%, SE = 0.04
Fear	0.82%, SE = 0.01	1.64%, SE = 0.01	15.16%, SE = 0.02	41.39%, SE = 0.03	54.10%, SE = 0.03	68.03%, SE = 0.04	68.03%, SE = 0.04	74.18%, SE = 0.03	70.90%, SE = 0.03	73.36%, SE = 0.04
Anger	22.13%, SE = 0.03	24.59%, SE = 0.03	31.15%, SE = 0.03	38.52%, SE = 0.03	47.54%, SE = 0.03	54.10%, SE = 0.03	57.79%, SE =0.02	63.93%, SE = 0.02	63.93%, SE = 0.02	67.21%, SE = 0.02
Disgust	2.87%, SE = 0.01	9.02%, SE = 0.02	18.03%, SE = 0.02	29.51%, SE = 0.03	54.51%, SE = 0.03	67.21%, SE = 0.04	71.31%, SE = 0.03	76.23%, SE = 0.03	79.51%, SE = 0.03	75.28%, SE = 0.04
Surprise	2.46%, SE = 0.01	5.74%, SE = 0.02	30.74%, SE = 0.02	50.41%, SE = 0.03	70.08%, SE = 0.03	81.15%, SE = 0.03	86.07%, SE = 0.03	87.70%, SE = 0.02	84.84%, SE = 0.03	88.52%, SE = 0.03
Average	9.02%, SE = 0.01	14.00%, SE = 0.01	26.30%, SE = 0.01	44.60%, SE = 0.02	59.29%, SE = 0.02	69.13%, SE = 0.02	73.63%, SE = 0.02	78.01%, SE = 0.01	76.64%, SE = 0.01	78.07%, SE = 0.02

Note: PAFs = P1vital® Affective Faces set. PoFA = Pictures of Facial Affect. SE = standard error of the mean.
